# Rethinking rotator cuff repair: a critical opinion on the “double pulley-triple row” technique

**DOI:** 10.3389/fsurg.2024.1494664

**Published:** 2024-12-23

**Authors:** Kyu-Cheol Noh, Sreejith J. Thampy, Vivek Kumar Morya

**Affiliations:** ^1^ Department of Orthopedic Surgery, Hallym University Dongtan Sacred Heart Hospital, Dongtan, Republic of Korea; ^2^ Department of Orthopedic Surgery, Hallym University Sacred Heart Hospital, Anyang, Republic of Korea

**Keywords:** double pulley-triple row (DPTR) technique, arthroscopy, rotator cuff repair, massive rotator cuff tear, suture bridge anchor fixation

## Introduction

Arthroscopic rotator cuff repair has become the standard for treating rotator cuff tears, valued for its minimally invasive nature, quicker recovery times, and favorable functional outcomes (1). However, despite its widespread use, the procedure continues to grapple with a significant challenge of high re-tear rates, particularly in cases involving larger tears or poor tendon quality (2). These re-tears not only compromise surgical success, but also negatively affect long-term patient outcomes. Addressing this challenge has become *a priori*ty in orthopedic surgery, driving the exploration of more effective and reliable techniques (3). While traditional approaches, such as single-row and double-row repairs, are widely used, they do not consistently eliminate re-tear risks, underscoring the need for innovative solutions. One such approach, the Double Pulley-Triple Row (DPTR) technique, which has been introduced in our clinic, was found to be a promising advancement aimed at overcoming these limitations.

The single-row technique, commonly employed for its simplicity and shorter operative time, involves anchoring the tendon to the bone using a single line of anchors (4). Although effective in some cases, this approach often fails to provide adequate footprint coverage or optimal tension distribution, particularly in larger tears (5). Consequently, re-tear rates for single-row repairs remain high, ranging from 20% to 40%, largely due to suboptimal biomechanical strength and incomplete tendon-bone integration (6). In contrast, double-row techniques have been developed to address these biomechanical deficiencies by increasing the tendon-to-bone contact area through anchors placed in both the medial and lateral rows. This approach improves force distribution and initial biomechanical strength, reducing retear rates by 10%–30% (6, 7). However, the added surgical complexity and cost have limited its widespread adoption, with studies showing mixed results regarding its superiority over single-row repairs.

To overcome the limitations of these conventional methods, hybrid approaches such as suture bridges and transosseous-equivalent repair have been developed (8). These techniques aim to combine the strengths of single- and double-row repairs by maximising tendon-bone contact and enhancing load sharing, particularly for larger or more complex tears (9). Preliminary evidence suggests that suture bridge techniques may reduce retear rates and improve repair strengths. However, challenges such as increased surgical complexity, higher costs, and limited long-term data have increased their acceptance (10).

The double pulley-triple row (DPTR) technique represents a novel solution designed to address the biomechanical and biological shortcomings of existing methods. By integrating a triple-row configuration, the DPTR technique enhances tendon-to-bone contact, distributes the load evenly, and improves mechanical stability (11). Its double-pulley mechanism allows for better tensioning and footprint coverage, potentially reducing retear rates. Early biomechanical studies suggested promising outcomes, although clinical validation in larger patient populations is necessary. This review examines the potential of the DPTR technique to revolutionise rotator cuff repair, evaluates its advantages over traditional approaches, and identifies areas for further research to optimise its clinical application.

## Understanding the traditional approaches

Historically, the gold standard for rotator cuff repair has been the single-row technique, which involves anchoring the torn tendon to the bone using a single row of sutures (12). While effective for smaller tears, this method often fails to fully restore the original footprint of the tendon, leading to uneven stress distribution and a significant risk of re-tear, particularly in larger tears (12, 13). To address these limitations, the double-row technique has emerged as a robust approach. By utilising two rows of sutures, this method increases the tendon-bone contact area and enhances biomechanical stability (14, 15). However, even with double-row repairs, significant challenges persist, particularly in cases of massive tears. Re-tears often occur near the musculotendinous junction (MTJ), where stress concentration and potential medial row strangulation can compromise repair (7, 16). Given these limitations, innovative techniques are continually being explored to optimise the outcomes of rotator cuff repair. The “double pulley-triple row” technique is a promising approach that aims to address the shortcomings of traditional methods (The techniques is accepted in the clinics in orthopaedic surgery CiOS). By incorporating specific design principles and surgical techniques, this method seeks to enhance tendon-bone healing, improve biomechanical function, and minimise the risk of retear.

## “Double pulley-triple row” technique

The Double Pulley-Triple Row (DPTR) technique is an advancement in arthroscopic methods designed to improve tendon fixation and promote superior healing outcomes in rotator cuff repair, particularly for large or massive tears (11). This innovative approach enhances tension distribution, increases tendon-bone contact, and bolsters structural stability, making it especially effective in cases where traditional techniques fall short. Below is a brief step-by-step description of the procedure (11).

### Step 1: Footprint medialization and medial row anchor placement

The patient was positioned laterally to optimise shoulder joint access for arthroscopy. In large or massive tears, the tendon often retracts from its original attachment site. Footprint medialization repositions the tendon closer to its anatomical footprint on the humerus, thereby restoring proper tension and alignment. After debriding the damaged tissue, medial row anchors were placed along the medial footprint to secure the tendon to bone. The sutures pass through the strongest section of the rotator cuff tendon, ensuring robust engagement with viable tissue for healing and resistance to postoperative stress.

### Step 2: Placement of the middle-row anchor

A middle-row anchor is placed just medial to the greater tuberosity to further lateralise the tendon and reduce tension on the repair. One limb of each medial row suture was passed through the tendon in a vertical pattern lateral to the medial sutures. This configuration distributes tension evenly and reduces strain on the musculotendinous junction. By aligning the tendon closer to its original footprint, this step improves the repair capacity to withstand mechanical loads and enhances the long-term stability.

### Step 3: Creation of the double-pulley system

**Figure 1:**
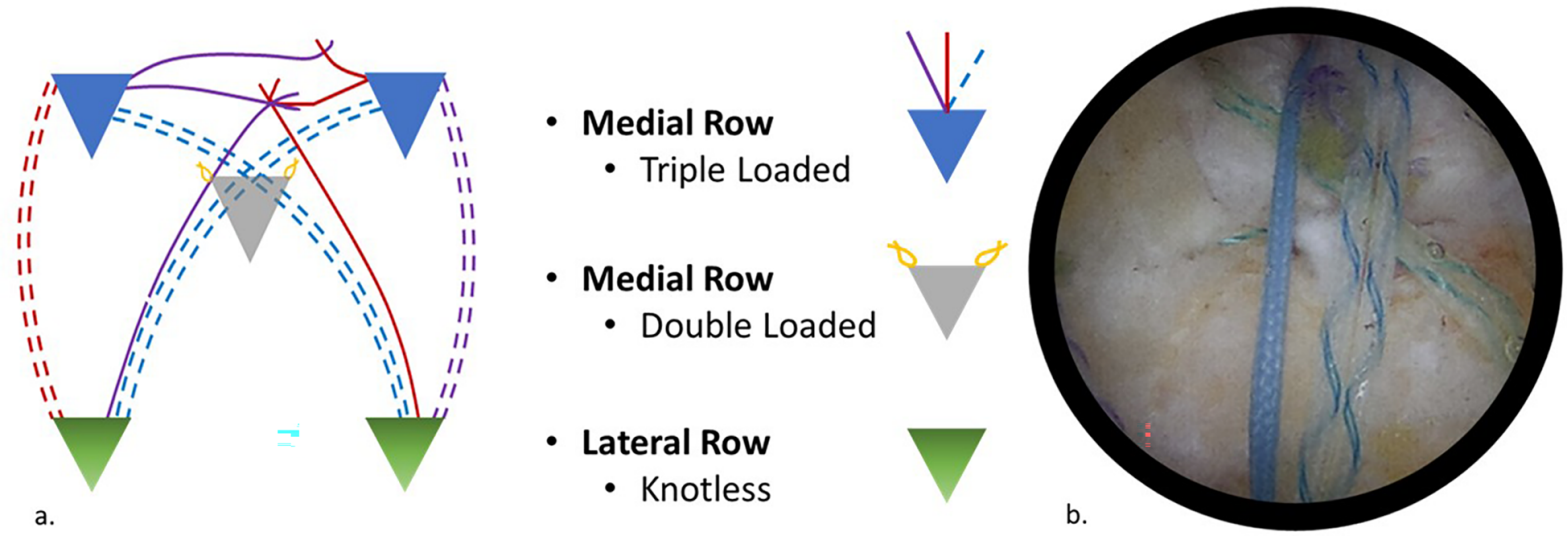
**(a)** Schematic diagram of construct. **(b)** Arthroscopic image using a 30-degree scope viewing through the lateral portal showing the final construct.

The double-pulley system is created to evenly distribute tension across the tendon. Sutures from the medial anchors were interconnected with static knots, and the remaining limbs were tied into sliding knots (Figure 1). This arrangement compresses the tendon against the bone without strangulation, reduces stress concentrations, and promotes a biologically favourable environment for healing.

### Step 4: Triple row rotator cuff repair

A lateral row of knotless anchors is placed along the greater tuberosity to complete repair (Figure 1). These anchors secure the remaining suture limbs, creating a broader footprint for enhanced biomechanical stability and reduced retear risk. This step promotes superior load distribution and tendon-bone integration, making the DPTR technique effective for complex tears.

## Discussion

The “double pulley-triple row” technique represents an innovative approach to arthroscopic rotator cuff repair, offering several biomechanical and clinical advantages. However, critical examination reveals that although this method introduces significant improvements over traditional techniques, it also raises questions regarding its cost, long-term outcomes, and biological implications (11).

The procedure, performed with the patient positioned laterally, begins with footprint medialization and the placement of a medial row of anchors. For massive tears, repositioning of the tendon closer to its anatomical footprint enhances the repair quality but adds complexity to the procedure. Anchoring the sutures through healthy cuff tissue ensures robust fixation; however, achieving this uniformly in all cases can be challenging, particularly in patients with poor tissue quality. The placement of the second and third rows of anchors, coupled with the pulley system, creates an expanded footprint and evenly distributes tension. This design aims to reduce stress concentrations, which are a primary cause of retears in traditional repairs. However, the assertion that the pulley system uniformly reduces tension must be substantiated through more rigorous biomechanical studies, as individual variability in tendon quality and repair site dimensions may influence outcomes (6, 7, 11).

The larger contact area between the tendon and bone is highlighted as a key advantage, enhancing the stability and mitigating the risk of tendon retraction. However, this benefit may not apply equally to all patients, particularly to those with compromised vascularity or significant degeneration. While the ability of the technique to minimise tendon strangulation and preserve vascularity is noteworthy, its biological implications warrant deeper investigation. Critics argue that emphasis on mechanical strength, evident in the addition of a third row of anchors, may inadvertently compromise the biological healing environment. Vascularity, which is crucial for tendon repair, can potentially be hindered by excessive mechanical manipulation, particularly in cases where the tissue quality is suboptimal. Although the double-pulley system aims to address this by evenly distributing tension, more comprehensive studies are needed to confirm its efficacy in promoting long-term tendon health.

The cost implications present another critical consideration. The inclusion of three rows of anchors inherently increases surgical expenses, which may deter widespread adoption. Although this extra cost is offset by reduced retear rates and better functional outcomes, this claim relies heavily on preliminary data. Long-term studies are necessary to validate these assertions, particularly when evaluating the economic viability of this technique across diverse healthcare systems. The potential reduction in revision surgeries is an important benefit, but its significance depends on the durability of the initial repairs, a factor yet to be conclusively demonstrated.

The risk of subacromial irritation due to knot placement in the double-pulley system, although considered minimal, cannot be overlooked. Although strategic anterior positioning of the knot minimises this risk, theoretical concerns remain. Subacromial irritation, even in isolated cases, can significantly affect patient outcomes and satisfaction. Thus, a long-term follow-up is essential to fully assess the safety and practicality of this knot configuration. Emphasis is needed to balance mechanical stability with biological healing. While preliminary data suggest that the “Double Pulley-Triple Row” technique achieves this balance, concerns persist about over-reliance on mechanical constructs. For example, the use of knotless anchors in the third row simplifies the procedure but may reduce the biological integration between the tendon and bone. The interplay between mechanical innovation and biological viability is complex and requires further research to optimise repair strategies.

Preliminary outcomes have shown promise, with patients demonstrating improved functional outcomes, faster recovery times, and lower retear rates than traditional double-row repairs. However, these findings are based on short-term follow-up and may not accurately reflect the long-term efficacy. Factors such as patient-specific characteristics including tear size, tissue quality, and comorbidities are crucial in determining the success of the technique. Additionally, the suitability of this method for addressing massive tears or poor-quality tissue remains a subject of ongoing investigation. Its application in these challenging cases underscores the need for further refinement to maximise both mechanical and biological outcomes.

Future research priorities include long-term follow-up studies to validate the durability of repairs, patient-specific applications to tailor the technique to individual needs, and biomechanical refinements to further optimise the tension distribution and minimise complications. The development of cost-effective alternatives, such as simplified anchor systems, could enhance the accessibility of this technique without compromising its efficacy. Furthermore, integration of advanced imaging and surgical planning tools may improve patient selection and procedural precision. The “Double Pulley-Triple Row” technique introduces significant advancements in rotator cuff repair, it is not without limitations. Its potential to reduce retear rates and enhance functional outcomes is encouraging; however, these benefits must be weighed against concerns regarding cost, long-term durability, and biological implications. Future research must address these challenges to establish this technique as a reliable standard for complex rotator cuff injuries. Comprehensive long-term studies are pivotal in determining whether this innovative approach can sustainably improve patient outcomes while remaining economically and biologically viable.

## Conclusion: this study represents a step forward in rotator cuff repair

In our opinion, the “double pulley-triple row” technique represents a significant advancement in rotator cuff repair. This technique offers a novel approach that can improve long-term patient outcomes by addressing the limitations of traditional single- and double-row repair. Its ability to provide greater mechanical stability while preserving the biological factors necessary for tendon healing makes it a promising option for surgeons seeking to reduce re-tear rates and improve patient recovery. While further research is needed to confirm its long-term efficacy, the early results of the “Double Pulley-Triple Row” technique are encouraging. Surgeons should consider incorporating this technique in their practice, particularly in patients with larger or more complex rotator cuff tears. As we continue to refine our approach to rotator cuff repair, the “Double Pulley-Triple Row” technique may become an essential tool in the surgical arsenal, helping to minimise re-tear rates and improve patient outcomes.
